# Identification of *Staphylococcus aureus* Causing Contamination in Raw Beef and Meat-Processing Environments in Ulaanbaatar, Mongolia

**DOI:** 10.1155/ijm/3806846

**Published:** 2025-01-30

**Authors:** Amgalanzaya Dorjgochoo, Anujin Batbayar, Altansukh Tsend-Ayush, Bayarlakh Byambadorj, Sarantuya Jav, Munkhdelger Yandag

**Affiliations:** ^1^Department of Molecular Biology and Genetics, School of Biomedicine, Mongolian National University of Medical Sciences, Ulaanbaatar, Mongolia; ^2^Department of Biomedicine, Etugen University, Ulaanbaatar, Mongolia; ^3^Department of Medicine, Global Leadership University, Ulaanbaatar, Mongolia

**Keywords:** antibiotic resistance, *eta*, *etb*, foodborne infection, *mecA*, raw beef meat, *S. aureus*, *sea*, *sed*, *tsst*, virulence genes

## Abstract

*Staphylococcus aureus* (*S. aureus*) is a Gram-positive bacterium capable of causing a range of infections and displaying significant antibiotic resistance. *S. aureus* can exhibit resistance to multi-antibiotics, particularly penicillin, methicillin, linezolid, and daptomycin. The prevalence of methicillin-resistant *S. aureus* (MRSA) ranges from 10%–50% in China and Russia, neighboring countries of Mongolia. This study aimed to assess *S. aureus* contamination in raw beef samples and surface swabs from meat-processing areas and markets, while detecting, as well as to detect virulence and resistance genes in the isolates. A total of 156 raw beef samples and 131 surface swabs were collected and analyzed using ISO 6888-1:2021 standards. The *nucA* gene specific to *S. aureus* was amplified by PCR, and antibiotic susceptibility was evaluated using the Kirby–Bauer disk diffusion method. Resistance genes (*mecA*, *mecC*, *vanA*, and *vanB*) and virulence genes (*sea*, *sed*, *tsst*, *eta*, and *etb*) were detected via PCR. The results showed contamination rates of 26.9% in raw beef and 15.3% in surface swabs. The isolates exhibited high resistance to oxacillin, ampicillin, and penicillin in meat samples and to oxacillin, tetracycline, azithromycin, and clindamycin in surface swabs. No resistance genes for vancomycin or methicillin (*mecC*, *vanA*, *vanB*) were detected. Virulence genes, including *tsst* (14.5%), *sea* and *etb* (9.7%), *eta* (3.2%), and *sed* (1.6%), were identified. Contamination was more prevalent in centers responsible for both transportation and sales, compared to meat-processing areas. These findings highlight the need for stricter hygiene and handling practices in meat transport and markets to reduce *S. aureus* contamination and limit the spread of resistant strains.

## 1. Introduction

Warm-blooded animals have Gram-positive *Staphylococcus* bacteria in their skin and mucous membranes [1]. Many tissue infections caused by *Staphylococcus*, including skin and soft tissue infections, pneumonia, endocarditis, osteomyelitis, mastitis, gastroenteritis, and toxic shock syndrome, can occur especially in humans and animals. It is a highly virulent bacterium that causes various diseases including foodborne illnesses and food poisoning [2, 3].

A total of 241,000 cases of staphylococcal food poisoning are recorded in the United States annually [4]. The disease of food poisoning originates from the transmission of consuming foods contaminated with *S. aureus* enterotoxins. Specifically, contaminated raw and processed meats, other animal-based products, eggs, milk, dairy products, and baked foods are frequently the source of the *S. aureus–*related infections [5]. Food products that are made by the meats of livestock are one of the sources of nutrients and contain high amounts of protein, minerals, and other health-promoting substances compared with plant-derived products [6]. Meat is easily infected with microbes and spoiled rapidly due to the high amount of water, proteins, minerals, biomolecules, and other nutrients, and it is also becoming a very comfortable condition for bacterial growth [7]. The frequency of *S. aureus* isolated in raw meat was found to be 1.3% in Nigeria [8], 15% in Egypt [9], 20.5% in the People's Republic of China [10], 26.31% in Iran [11], 27.8% in the United States [12], 29.4% in Algeria [13], 34.3% in Ethiopia [14], 35.4% in Korea [15], 32.8% in Japan [16], 40.38% in Morocco [17], 45% in Ghana [18], 46% in Colombia, 63% in Georgia [19], and 68% in Poland, respectively [20].

*S. aureus* can induce food poisoning due to its resistance to high temperatures and acidic environments, and it possesses numerous toxic components such as enterotoxins, acute toxic shock syndrome toxin, and exfoliative toxins [21, 22]. Because of the loss of hygienic safety, the isolates containing many types of toxins and sensitive to temperature can contaminate the food that is ready to consume and infect humans [23].

Antibiotic-resistant bacteria are a serious public health concern that is spreading globally. It exhibits that some bacteria are becoming resistant to antibiotics, which are medicines used to treat bacterial infections. This makes it harder to treat infections and can lead to more severe illnesses and even death. It is important for people to understand the risks of antibiotic resistance and take steps to prevent it, such as using antibiotics only when necessary and as prescribed by a healthcare professional.

Staphylococcal infections are typically treated with beta-lactam antibiotics; however, an increasing number of *S. aureus* isolates are resistant to beta-lactam antibiotics due to the excretion of beta-lactamases [24]. Methicillin is a semisynthetic antibiotic that was discovered in 1960 and is used to treat *S. aureus* infections. However, methicillin resistance happens when *mecA* combines with another deoxyribonucleic acid (DNA) part, making beta-lactam antibiotics less effective [25]. 20%–40% of *S. aureus* isolated from pork were found to be methicillin-resistant *Staphylococcus aureus* (MRSA) according to studies conducted in the Netherlands and Canada [26]. Currently, *S. aureus* isolate is well-recognized that it is highly resistant to penicillin and methicillin, and new antibiotics such as linezolid and daptomycin are becoming more effective due to the antibiotic resistance mechanisms [27].

Currently, *Staphylococcus aureus* is widely known for its high resistance to penicillin and methicillin; however, newer antibiotics such as linezolid and daptomycin are becoming increasingly effective in treating infections due to their ability to overcome specific antibiotic resistance mechanisms [28].

The prevalence of the MRSA isolate is estimated to be very high, ranging from 10% to 50%, in China and Russia, both of which border Mongolia.

*S. aureus* contamination was identified in 35% of raw beef meat samples collected from retail markets in Ulaanbaatar, Mongolia. The detected *S. aureus* isolate harbored various virulence genes, including enterotoxins (*sed, see*), toxic shock syndrome toxin (*tsst)*, and exfoliative toxins *(eta, etb*). Furthermore, the *mecA* gene, responsible for encoding the penicillin-binding protein 2A (PBP2A), was present in these isolates [29].

The purpose of our study is to determine *S. aureus*–associated contamination and detect its virulence genes in samples of raw beef meat and surface smears collected from meat-processing areas and meat markets.

## 2. Material and Method

### 2.1. Study Design and Sample

The study employed a cross-sectional design conducted from October 2022 to April 2023, systematically collecting samples from meat market areas where meat is processed and sold. The focus was on two primary locations: the general slaughterhouse at Emeelt and nearby food markets, located approximately 25 km from the slaughterhouse, known for openly displaying meat for a significant portion of Ulaanbaatar's consumers. Surface samples were meticulously gathered from contact points on beef, ensuring direct interactions between the meat and surfaces were targeted. These samples were placed in sterile, single-use ziplock bags, labeled for accurate identification. In addition to surface samples, swabs were taken from various sites within the meat-selling environments, including sellers' hands, gloves, aprons, and environmental surfaces such as meat counters, racks, scales, and carts. To maintain consistency, sterile cotton swabs soaked in 10 mL of brain heart infusion (BHI) broth were utilized for all swab samples. The distribution of surface swab samples included 32 from the hands of butchers and vendors, 30 from meat hangers, 22 from platforms, 14 from scales, 11 from knives, eight from aprons, eight from meat racks and carts, and six from hanger poles. All collected samples were promptly transported to the microbiology laboratory at the Mongolian National University of Medical Sciences where they were processed within 2 hours of collection to mitigate the risks of degradation or contamination.

### 2.2. Isolation and Identification of *S. aureus* From Meat Samples

To detect *S. aureus* in the collected meat samples, 25 g of each sample was placed in 225 mL of peptone broth and homogenized. One milliliter of the homogenized solution was then diluted by a factor of 10^3^, in accordance with ISO 6888-1:2021. Subsequently, 1 mL of the diluted solution was evenly spread onto Baird–Parker (BP) selective medium (Biolab, Hungary) which is designed to support the growth of *S. aureus* and distinguish it from other bacteria based on colony morphology. The plates were incubated at 37°C for 24 h. The standard *S. aureus* isolated ATCC 25923 was utilized as a control in the experimental procedures, ensuring consistency and reliability in the comparative analyses. After incubation, black and shiny colonies with white and clear zones were identified as suspected *S. aureus*. Up to five colonies were selected for further identification using polymerase chain reaction (PCR) to confirm the *S. aureus* isolate.

### 2.3. Identification of *S. aureus* Using PCR Assay

DNA was extracted from a bacterial suspension prepared from the cultured isolates. The suspension was boiled at 100°C for 10 min, followed by centrifugation at 12,000 rpm for 10 min to sediment the cellular debris. The concentration and purity of the extracted DNA were measured using a nanodrop spectrophotometer (Thermo Fisher Scientific, LLC) before proceeding with further analysis.

The specific identification of *S. aureus* was performed by amplifying a 270-bp product of the *nucA* gene, which encodes the *S. aureus*–specific thermonuclease, via PCR. The PCR mixture included 0.5 μL (100 pmol/μL) of each primer, nuc-F (5′-GCGATTGATGGTGATACGGTT-3′) and nuc-R (5′-AGCCAAGCCTTGACGAACTAAAGC-3′) [30, 31], along with 2 μL of the extracted DNA, and was combined with the PCR master mix (Bioneer, Korea) to a final volume of 25 μL.

The PCR amplification was carried out with an initial denaturation at 95°C for 10 min, followed by 37 cycles of denaturation at 94°C for 1 min, annealing at 55°C for 30 s, and extension at 72°C for 1.5 min. A final extension step was performed at 72°C for 10 min. The resulting PCR products were visualized by electrophoresis on a 1.5% agarose gel run at 100 V for 30 min and stained with ethidium bromide for 20 min [28].

### 2.4. Detection of *S. aureus* Virulence and Antibiotic Resistance Genes by PCR

The detection of *S. aureus* virulence genes, including enterotoxin genes (*sea, sed*), toxic shock syndrome toxin gene *(tsst*), and exfoliative toxin genes (*eta, etb*), was performed using multiplex PCR in two separate sets, as described in the literature [32]. The primers utilized for the PCR amplification are detailed in Table 1.

The PCR steps were carried out as follows: 94°C for 5 min, 94°C for 2 min, 55°C for 2 min, 72°C for 2 min, and 72°C for 7 min.

Table 2 shows the sequence of primers used to detect *mecA* and *mecC* genes encoding PBP2A, and *vanA* and *vanB* genes encoding vancomycin resistance proteins. The PCR conditions are shown in Table 3.

Amplified PCR products were detected by running a 1.5% agarose gel at 100 V for 30 min and stained with ethidium bromide for 20 min.

### 2.5. Antibiotic Susceptibility Testing

Antibiotic susceptibility of the isolates was assessed using the disk diffusion method on Mueller–Hinton agar (Difco, Franklin Lakes, NJ, USA). Each isolate was tested against a panel of antibiotics, including ampicillin (10 μg), oxacillin (30 μg), gentamicin (10 μg), tetracycline (30 μg), chloramphenicol (50 μg), penicillin (10 μg), clindamycin (2 μg), azithromycin (15 μg), and ciprofloxacin (5 μg) (Biolab, Budapest, Hungary). The inoculated plates were incubated at 37°C for 24 h, followed by measurement of the inhibitory zone diameters. Results were compared to the criteria set forth by the Clinical and Laboratory Standards Institute (CLSI) guidelines (M100-S27) for interpretation. The standard *S. aureus* isolate ATCC 25923 was employed for validating the antibiotic discs.

### 2.6. Statistical Analysis

The statistical analysis for this study was conducted using IBM SPSS Statistics version 25. Fisher's exact test and the chi-square test were employed to analyze the data. A *p* value of < 0.05 was considered to indicate statistical significance.

## 3. Results

A total of 156 raw beef meat and 131 surface swab samples were collected and analyzed in the study. The 26.9% (42/156) of raw beef meat samples and 15.3% (20/131) of surface swabs were contaminated with *S. aureus* isolate.

**Figure 1:**
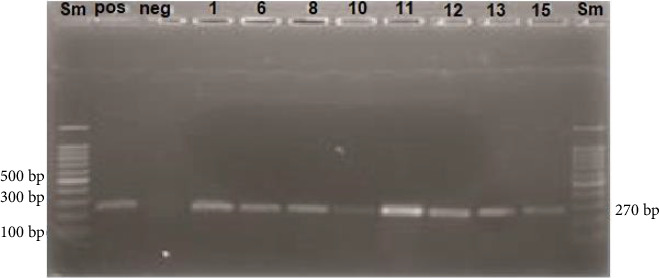
Identification of *nucA* gene: The size marker used is a small molecular weight DNA ladder (100 bp). The positive control is a known sample containing the *nucA* gene, and the negative control is nuclease-free distilled water. The *nucA* gene product is identified at 270 bp.

During the study, the *nucA* gene was identified using a small molecular weight DNA ladder (100 bp). A known sample containing the *nucA* gene served as the positive control, while nuclease-free distilled water was used as the negative control. The *nucA* gene product, measuring 270 bp in length, was detected in the samples analyzed, confirming the presence of the gene (Figure 1).

In addition, contamination with *S. aureus* isolate was detected in 31.3% (41/131) of raw beef meat samples and 17.3% (14/81) of surface swab samples collected from 12 commercial centers, where it was transported within 25 km from the meat-processing area. Moreover, it was also detected in 4% (1/25) of beef and 12% (6/50) of surface swab samples collected from meat-processing areas. Although the bacterial contamination was detected in both meat market and meat-processing area samples, it was detected 8 times higher in the beef samples from the meat market than that in the meat-processing area. Also, *S. aureus* contamination was 1.4 times higher in surface swabs from the meat market than that in the meat-processing area (*p*=0.046). It was not detected in 50 samples of raw beef meat collected from the meat-processing area and meat market. In the meantime, the contamination of *S. aureus* was detected in three samples of surface swabs (Table 4).

As regard to the type of surface swab samples used in this study, the samples were collected from different locations including 32 from the hands of butchers and vendors, 30 from meat hangers, 22 from platforms, 14 from scales, 11 from knives, eight from aprons, eight from meat racks and meat carts, and six surface swabs from hanger poles. Upon analysis, *S. aureus* isolates were found in 27% (8/30) of swabs taken from the meat rack, 25% (2/8) of the apron, 21.9% (7/32) of the seller's hands, and 18.2% of the knives (2/11) and 12.5% (1/8) of the meat carts (Table 5).

Antimicrobial susceptibility testing of the 62 isolates showed resistance to the majority of tested antibiotics: 87.1% (*n* = 54) for oxacillin, 69.4% (*n* = 43) for ampicillin, 61.3% (*n* = 38) for penicillin, 45.2% (*n* = 28) for clindamycin, 40.3% (*n* = 25) for azithromycin, 38.7% (*n* = 24) for tetracycline, 24.2% (*n* = 15) for ciprofloxacin, 16.1% (*n* = 10) for gentamicin, and 16.1% (*n* = 10) for chloramphenicol. Hence, it can be concluded that *S. aureus* isolates detected in raw beef meat samples were highly resistant to the antibiotics including oxacillin, ampicillin, and penicillin (*p* ≤ 0.05) (Table 6).

However, more than half of the isolates detected in surface swab samples were resistant to oxacillin, tetracycline, azithromycin, and clindamycin.

Five virulence genes and four antibiotic resistance genes were investigated in our study. The *vanA, vanB*, and *mecC* genes were not detected among all isolates. However, the *mecA* gene encoding PBP2A was detected in 17 (27.4%) isolates. Concerning the virulence genes, the *tsst* gene encoding toxic shock syndrome toxin was harbored by nine (14.5%) isolates, and the *etb* gene encoding exfoliative toxin Type B in six (9.7%) were detected among all *S. aureus* isolates collected from the beef. Moreover, eta gene for exfoliative toxin A and sed gene for enterotoxin D were detected in 3.2% (*n* = 2) and 1.6% (*n* = 1) among all the isolates, respectively. Of the 7 methicillin-resistant isolates, isolated from surface swabs, three isolates were detected among the samples of hands, three of meat hangers, and two of aprons, while one isolate with *mecA* and *tsst* genes was detected in hand swab samples (Table 7).

This table presents the detection rates of infection-related virulence genes in *Staphylococcus aureus* isolated from 62 samples. Among the samples, 53.2% showed no detectable virulence genes, while 32.3% had one virulence gene, 11.3% exhibited two genes, and 1.6% displayed three or four genes, respectively. Notably, in specimens with two or more virulence genes, the *mecA* gene was frequently codetected with other virulence factors, suggesting a heightened pathogenic potential combined with antibiotic resistance. The *mecA* gene, which confers methicillin resistance, was identified nine samples, with 88.9% of these originating from raw beef specimens (Table 8).

## 4. Discussion

Every country consumes large quantities of meat and animal products high in fat, protein, and vitamins. As the world's population has doubled in the last 50 years, the amount of meat products consumed has tripled. There are over 60 million domestic animals registered in Mongolia. Animal-derived products, particularly meat, are mostly consumed for daily meals [40]. Therefore, meat consumption is high in Mongolia, and it ranks 10th in the world and 2.7 times higher than the average of the world [41].

In this study, *Staphylococcus aureus* contamination was detected in 21.6% of the total raw beef meat samples collected from Ulaanbaatar. Of these, 4% were from meat-processing areas, while 32.1% originated from meat markets. Compared to findings from other nations—such as Iran (26.31%) [11], USA (27%–28%) [12], Algeria (29.5%) [13], Japan (32.8%) [16], Ethiopia (34.3%) [14], and Korea (35.4%) [15]—our results are on the lower end of the spectrum. However, contamination rates in Nigeria (1.3%) [8], Egypt (15%) [9], and China (20.5%) [10] were lower than that in our study. In contrast, studies from Morocco (40.38%) [17], Ghana (45%) [18], Colombia (46%), Georgia (63%) [19], Poland (68%) [20], and Denmark (68%) [42] reported higher contamination levels. These results suggest that contamination rates of meat vary significantly across countries.

This disparity between the processing and retail environments supports previous studies that point to noncompliance with hygiene standards and poor sanitation practices as major factors contributing to contamination in the supply chain. In our study, surface swab samples collected from butcher and vendor hands (21.9%) and meat hangers (27%) showed the highest rates of contamination, suggesting that improper handling and unhygienic conditions in retail settings contribute significantly to *S. aureus* contamination. Inadequate sterilization of equipment such as knives (18.2%) and aprons (25%) further exacerbates the issue, highlighting the need for improved sanitation practices in these areas.

Swab samples collected from meat markets and processing areas were analyzed for *Staphylococcus aureus* contamination, indicating a contamination rate of 15.3%. Contaminated samples included swabs taken from both workers' hands and meat-hanging hooks. While efforts are made to maintain cleanliness at counters and work surfaces, inadequate cleaning and disinfection of meat-hanging racks can lead to contamination, as bacteria from one piece of meat can spread to the next. This highlights the importance of proper sanitation and surface cleaning in preventing contamination. Food manufacturers must prioritize food safety by maintaining the cold chain and ensuring all surfaces are thoroughly cleaned and disinfected.

The global rise of antibiotic-resistant bacteria poses a major challenge to human health, veterinary care, and food safety. These bacteria can cause severe infections, leading to longer hospital stays, higher mortality rates, and increased medical costs. After the identification of livestock-associated (LA)-MRSA of animal origin in 2005, the issue of antibiotic-resistant bacterial contamination in food products has gained worldwide attention [43].

In this study, the disk diffusion method was used to assess the antibiotic resistance of *S. aureus* isolates. Of these, 87.1% were resistant to ampicillin, 69.4% to oxacillin, 61.3% to penicillin, and 16.1% to chloramphenicol. Our findings align with studies from Ethiopia, where 92.73% of isolates were resistant to penicillin, and 74.5% were resistant to cefoxitin, with minimal vancomycin resistance [14]. Similarly, a Turkish study found that over 50% of isolates were resistant to ampicillin and penicillin. The widespread use of penicillin and other *β*-lactam antibiotics in treating both human and animal infections likely contributes to the high levels of resistance observed in meat [16]. This suggests that contamination in retail environments may not only involve more resistant strains but also reflect inadequate sanitation and disinfection practices in these settings.

A growing public health concern is the emergence of highly virulent, methicillin-resistant bacteria, particularly those resistant to *β*-lactam antibiotics, which contaminate animal products and infect humans and animals. In this study, the *mecA* gene, which confers resistance to methicillin, was detected in 27.4% of isolates, while the *mecC* gene was not found. This is consistent with findings from Egypt, where *mecA* and *mecC* genes were detected in only 4.2% of raw beef isolates [44]. Moreover, no vancomycin resistance genes (*vanA or vanB*) were detected in our study.

Regarding virulence genes, we identified the following toxin-encoding genes among the *S. aureus* isolates isolated from beef*: tsst* (toxic shock syndrome toxin) in 14.5%, *etb* (exfoliative toxin B) in 9.7%, *sea* (staphylococcal enterotoxin A) in 9.7%, *eta* (exfoliative toxin A) in 3.2%, and *sed* (staphylococcal enterotoxin D) in 1.6%. Comparatively, in other studies*, sed* was found in 3.4% of isolates from China [10], *sea* in 30.3% of isolates from Italy, and *tsst* in 15.8%–40% of isolates from Spain [45]. These differences in the expression of virulence factors may be attributed to variations in contamination pathways, transmission hosts, and other risk factors associated with *S. aureus* contamination in beef.

*Staphylococcus aureus* can contaminate raw meat at various stages of processing, from infected animals at slaughter to improper skinning, cleaning, storage, and distribution. Noncompliance with hygiene standards, unhygienic conditions in slaughterhouses, and inadequate transportation and equipment sterilization may all contribute to contamination [14]. The higher contamination rates and resistance profiles observed in the retail stage highlight the need for targeted interventions. Strengthening hygiene controls during meat transportation and storage, as well as implementing stricter sanitation protocols in retail environments, could help reduce the risk of contamination. Furthermore, future research should focus on identifying specific risk factors that contribute to *S. aureus* contamination and foodborne illness outbreaks to develop more effective food safety strategies.

## 5. Conclusion

This study highlights significant public health risks from *Staphylococcus aureus* contamination in Mongolia's beef supply chain. A total of 156 raw beef meat and 131 surface swab samples were collected, showing contamination in 26.9% of raw beef meat and 15.3% of surface swabs. Contamination was notably higher in meat-selling areas than in meat-processing areas, with beef samples from markets showing 8 times more contamination. Antimicrobial resistance testing revealed that *S. aureus* isolates were highly resistant to antibiotics, especially oxacillin, ampicillin, and penicillin. The *mecA* gene, which confers methicillin resistance, was detected in 27.4% of isolates, and several virulence genes were also found, increasing the bacteria's pathogenicity. The findings emphasize the need for stricter hygiene measures and better control in meat processing, transportation, and retail environments to reduce contamination and antibiotic-resistant strains in the food supply chain.

## 6. Limitation

The study's findings may be somewhat limited in generalizability, as the sample collection was confined to specific locations in Ulaanbaatar, such as the Emeelt slaughterhouse and nearby food markets, which may not fully represent the broader diversity of meat markets or processing facilities across the country. Additionally, the focus on beef exclusively limits the scope of the results; extending similar analyses to other types of meat, such as poultry, pork, or mutton, could provide a more comprehensive perspective on *S. aureus* contamination across the meat industry in Ulaanbaatar.

## Figures and Tables

**Table 1:** Primers for the amplification of Staphylococcal genes.

Genes	Sequences (5′-3′)	Size of amplicon (bp)	Set	Reference
*sed*	F-CAATATAGGAGAAAATAAAAG R-ATTGGTATTTTTTTTCGTTC	278	I	[30]
*eta*	F-GCAGGTGTTGATTTAGCATT R-CTGGTGAAGTTGTAATCTGG	93		[33]
*etb*	F-GGTTATCAATGTGCGGGTGG R-GTTTTGGTGCTTCTCTTG	226	II	
*sea*	F-GGTTATCAATGTGCGGGTGG R-CGGCACTTTTTTCTCTTCGG	102		[30, 34]
*tsst*	F-ACCCCTGTTCCCTTATCATC R-TTTTCAGTATTTGTTAACGCC	326		[35]

**Table 2:** Primer sequences used to detect antibiotic resistance gene.

Gene	Sequences (5′-3′)	Size of amplicon (bp)	Reference
*mecA*	F-ACTGCTATCCACCCTCAAAC R-CTGGTGAAGTTGTAATCTGG	163	[36]

*mecC*	F-TGAACGAAGCAAGAGTACACC R-AGATCTTTTCCGTTTTCAGCCT	238	[37]

*vanA*	F-GGCAAGTCAGGTGAAGATG R-ATCAAGCGGTCAATCAGTTC	713	[38]

*vanB*	F-GTGACAAACCGGAGGCGAGGA R-CCGCCATCCTCCTGCAAAAAA	430	[39]

**Table 3:** Primer reaction conditions for PCR detection of antibiotic resistance genes.

Gene	*mecA*	*mecC*	*vanA*	*vanB*
Initial denaturation	94°C 5 min	94°C 5 min	94°C 5 min	94°C 10 min

Denaturation	94°C 2 min	94°C 30 s	94°C 1 min	94°C 30 s

Annealing	55°C 2 min	59°C 1 min	55°C 1 min	50°C 45 s

Extension	72°C 2 min	72°C 1 min	72°C 2 min	72°C 30 s

Final extension	72°C 7 min	72°C 10 min	72°C 5 min	72°C 10 min

Cycle	30	30	40	30

**Table 4:** Raw beef meat and surface swab sample contamination.

Sample type	Meat-processing area		Meat market		Total	
	Revealed % (*n*)	Not revealed % (*n*)	Revealed % (*n*)	Not revealed % (*n*)	Revealed % (*n*)	Not revealed % (*n*)
Beef meat	4 (1)	96 (24)	31.3 (41)	68.7 (90)	21.6 (42)	78.4 (114)
Surface swab	12 (6)	88 (44)	17.3 (14)	81.7 (67)	15.3 (20)	84.7 (111)

**Table 5:** Prevalence of *Staphylococcus aureus* on various surface swab locations in a meat-processing environment.

Surface swab location	Total samples	Positive for *S. aureus*	
	*N*	*n*	%
Hands of butchers and vendors	32	7	21.9
Meat hangers	30	8	27
Platforms	22	—	—
Scales	14	—	—
Knives	11	2	18.2
Aprons	8	2	25
Meat racks	8	1	12.5
Meat carts	8	1	12.5
Hanger poles	6	—	—

**Table 6:** Antibiotic resistance of *S. aureus*.

Antibiotic	Raw beef meat *n* (%)	Surface swab *n* (%)	Total *n* (%)
Ampicillin	38 (90.5)	5 (25)	43 (69.4)
Oxacillin	38 (90.5)	16 (80)	54 (87.1)
Gentamicin	8 (19)	2 (10)	10 (16.1)
Tetracycline	12 (28.6)	12 (60)	24 (38.7)
Chloramphenicol	5 (11.9)	5 (25)	10 (16.1)
Penicillin	34 (81)	4 (20)	38 (61.3)
Clindamycin	16 (38.1)	12 (60)	28 (45.2)
Azithromycin	13 (31)	12 (60)	25 (40.3)
Ciprofloxacin	12 (28.6)	3 (15)	15 (24.2)

**Table 7:** Occurrence of *mecA* gene and virulence genes.

Virulence genes of *S. aureus*	Raw beef meat samples *n* (%)	Surface swab *n* (%)	Total *n* (%)
*mеcA*	10 (23.8)	7 (35)	17 (27.4)
*sed*	1 (2.4)	No detected	1 (1.6)
*eta*	2 (4.8)	No detected	2 (3.2)
*etb*	6 (14.3)	No detected	6 (9.7)
*sea*	6 (14.3)	No detected	6 (9.7)
*tsst*	8 (19)	1 (5%)	9 (14.5)
Total	33 (80.5)	8 (19.5)	41 (100)

**Table 8:** Overlapping expression of virulence genes in *S. aureus*.

Number of virulence genes coexpressed	*n* (%)	Combination of genes
Undetected	33 (53.2)	
1 gene detected	20 (32.3)	
2 gene detected	7 (11.3)	*mecA + tsst; mecA* + *sea*
3 gene detected	1 (1.6)	*mecA + etb + sea*
4 gene detected	1 (1.6)	*mecA + eta + etb + sea*
Total	62 (100)	

## Data Availability

The data used to support the findings of this study are available from the corresponding author upon reasonable request.
